# Protection motivation when using biocidal products – A survey study in Germany

**DOI:** 10.1007/s11356-024-34639-2

**Published:** 2024-08-07

**Authors:** Angela Bearth, Livia Thoma, Christiane Stark, Michael Siegrist

**Affiliations:** 1 Consumer Behavior, Institute for Environmental Decisions (IED), ETH Zurich, CHN J 75.2, Universitaetstrasse 22, 8092 Zurich, Switzerland; 2 German Environment Agency, Section Biocides (IV 1.2), Woerlitzer Platz 1, 06844 Dessau-Roßlau, Germany; 3 Swiss Centre for Applied Human Toxicology (SCAHT), University of Basel, Basel, Switzerland

**Keywords:** Biocidal products, Regulation, Risk management, Risk perception, Protection motivation theory, Locus of control

## Abstract

Due to their inherent properties, biocidal products might pose a risk to human and animal health and the environment. In risk management, there exists uncertainty about private users' comprehension of and willingness-to-adhere to use instructions that mitigate these risks (e.g., limit frequency of use or application area). This study aimed at providing insights into the users' perspective by focusing on their attention, comprehension, and the attitudinal predictors of protection motivation. In the online study (*N* = 957 participants from Germany) were introduced to a hypothetical purchase scenario featuring an insect spray and a realistic set of use instructions. Next, the participants' attention for the use instruction was measured in self-report and by tracking the time spent on the page with the use instruction, while Likert scale-type questions measured the comprehensibility, protection motivation, and predictors. Participants reported higher protection motivation if they spent more time with the use instruction, perceived it as comprehensible, rated the threat to humans, animals, and the environment as more severe, perceived themselves as responsible (i.e., internal locus of control) and capable (i.e., self-efficacy) of adhering to the use instructions, perceived adherence to the use instructions as effective (i.e., response efficacy) and did not perceive biocidal products as inherently safe (i.e., neutral locus of control). These results offer valuable information for an improved regulation of biocidal products and better management of potential risks associated with their use. They also provide concepts for interventions to ensure users of biocidal products follow the instructions for a safe use and better protection of the environment.

## Introduction

Within the Biocidal Products Regulation (BPR, Regulation (EU) 528/2012), biocides are defined as products, which are used to protect human and animal health as well as natural or manufactured materials against harmful organisms like pests or bacteria (European Union 2012). Biocidal products can be grouped, according to their intended use, into four main groups: disinfectants, preservatives, pest control, and other biocidal products (i.e., antifouling, and embalming fluids). Due to their inherent properties, these products might pose a risk to human and animal health and the environment (Kristiansson et al. 2021). One major principle laid down in the BPR (European Union 2012) is that only biocidal products with no unacceptable risks to human health, animal health and environment shall be authorised and placed on the market. For biocidal products for which potential risks were identified during the risk assessment, the BPR foresees the assignment of risk mitigation measures as a central instrument for risk management. Risk mitigation measures range from restriction of user category, restriction of application area to specific requirements for a safe application (e.g. limited number of use per season) (Berger-Preiß et al. 2005; Berger-Preiß et al. 2009; European Union 2012).

However, the allocation of risk mitigation measures as a legal precondition for the authorisation of many biocidal products denotes a point of uncertainty in risk management: Will users correctly follow these instructions? This point of uncertainty introduces a strong need for insights from the user perspective. Unfortunately, the literature base on this is sparse with literature mostly targeting other household chemicals (Basso et al. 2016; Bearth et al. 2020). While some transfers to biocidal products can be made from these insights, there exists a lack of studies on users' comprehension of and attention for use instructions on biocidal products and their motivation to protect human and animal health and the environment. This study aimed at adding to this literature base and providing insights for regulation and the industry on users' interactions with biocidal products. Two theoretical frames were used and integrated for this study: the Protection Motivation Theory (Maddux and Rogers 1983; Milne et al. 2000; Prentice-Dunn and Rogers 1986; Rogers 1975) and the construct of Locus of Control (Strudler Wallston and Wallston 1978).

## Theoretical background

Primarily three sequential aspects are relevant when thinking about the users' perspective of biocidal products and use instructions: 1) attention, 2) comprehension, and 3) protection motivation. The previous literature relevant for these three aspects is presented and discussed below.

First, users must not only be exposed to the use instructions on biocidal products but direct their attention to these use instructions. Prior literature suggests that this is more likely the case if products are spontaneously judged to be dangerous or highly effective (Bearth et al. 2020; Buchmueller et al. 2020). This judgment can be based on prior knowledge and experiences, but also on cues from the product packaging that might or might not provide the user with objective risk information (Bearth et al. 2017; Buchmueller et al. 2022). In a study focused on warnings on cleaning products (Geuens et al. 2021), users focused on average only 30 s of their attention on the warnings even though they were instructed to inspect them closely. Generally, literature suggests short attention spans for objective risk information on household chemicals due to habit, familiarity, or low salience of warnings (Bearth et al. 2020; Buchmueller et al. 2020, 2022). Regarding biocidal products, it is to be expected that some products, such as treatments against pests, will raise high initial risk perceptions and thus, activate higher levels of attention than other household chemicals. However, consumers might not always be aware of the biocidal properties of the products they use and thus, these products might garner low risk perception and thus, low attention from consumers (Wieck et al. 2018a, 2018b).

Second, users must process and comprehend the information provided to them. To our knowledge, no prior literature exists on the comprehensibility of use instructions on biocidal products, as most literature focuses on comprehensibility of warning symbols and pictograms (Bearth et al. 2020; Boelhouwer and Davis 2010; Boelhouwer et al. 2013; Dugger-Webster and LePrevost 2018; Geuens et al. 2021). From other areas (e.g., medicine labels), however, it is known that comprehensibility is positively impacted by the clarity of phrasing, conciseness of writing, vivid examples, and accessible terminology (Fuchs and Hippius 2007; Maghroudi et al. 2021).

Third, users must be motivated to adhere to the use instructions and thus, protect human and animal health and the environment. Even if users pay attention to the use instruction and comprehend them, low protection motivation leads to non-compliance and thus, might imply risks to health and the environment. Extensive research shows that consumers sometimes fail to recognise the personal relevance of a warning or the risk that a household chemical might pose to health or the environment (Bagheri et al. 2021; Bearth et al. 2020; Patel et al. 2008; Riley et al. 2001). To understand users' protection motivation, it might be useful to turn to the health psychology and behavioural change literature. Within this branch of research, various frameworks and empirical models were developed for predicting intention (i.e., the intent to engage in or refrain from a particular behaviour, such as smoking) and behaviour (i.e., self-reported or exhibited behaviour) based on several determinant variables or different awareness and motivational stages. Intention is sometimes conceptualised as a precursor to behaviour, but has also been criticised for their small to medium predictive power due to the limitations of self-report and the influence of individual and contextual factors (e.g., norms, habits, impulse control) (Ajzen 2011; Sheeran 2002; Sheeran and Webb 2016). To choose an appropriate theory and variable of interest, it is recommended to consider the context (Ajzen 2011).

The Protection Motivation Theory (PMT) investigates responses to potential threats and when faced with specific warning messages (Prentice-Dunn and Rogers 1986; Rogers 1975). Due to this focus on risk, it is applicable to the context of biocidal products. The PMT focuses on its core variable "Protection Motivation," which denotes the willingness to take measures to protect the own health, the health of others, animals or the environment. This core variable is predicted by the concepts of "Threat Appraisal" and "Coping Appraisal." The former includes the perceived likelihood and severity of the threat, while the latter denotes the person's perceived ability to cope with the threat. Threat and coping appraisal impact a person's protection motivation. This means that a high threat appraisal and a high coping appraisal is associated with higher protection motivation, whereas a low threat appraisal and a low coping appraisal is associated with low protection motivation. It has also been suggested that a high threat appraisal and a low coping appraisal might lead to a maladaptive response and low protection motivation (Maddux and Rogers 1983; Milne et al. 2000; Shillair 2020). Another concept that might be of relevance for people's responses to warnings on biocidal products is "Locus of Control," which describes to whom a person attributes control regarding a specific threat (Atshan et al. 2020; Rotter 1966; Strudler Wallston and Wallston 1978; Tosun et al. 2020). The locus of control can either be internal (i.e., the user is in control of the threat that a biocidal product might pose), external (i.e., someone other than the user is in control of the threat that a biocidal product might pose), or neutral (i.e., nobody is in control). It is assumed that a high internal, and low external and low neutral locus of control would result in higher protection motivation.

To conclude: research that aimed at understanding the users' interactions with chemical products has up to now primarily focused on the first two aspects: attention and comprehension (Bearth and Siegrist 2019; Boelhouwer and Davis 2010; Buchmueller et al. 2022; Geuens et al. 2021). There is a need to focus on all three aspects introduced above, namely the users' attention, comprehension, and the attitudinal predictors of protection motivation. This can help uncover the most important barriers and drivers of the safe use of a biocidal product and can thus, inform product and label design and future biocidal regulation and policy.

## Study goals and research questions

**Fig. 1:**
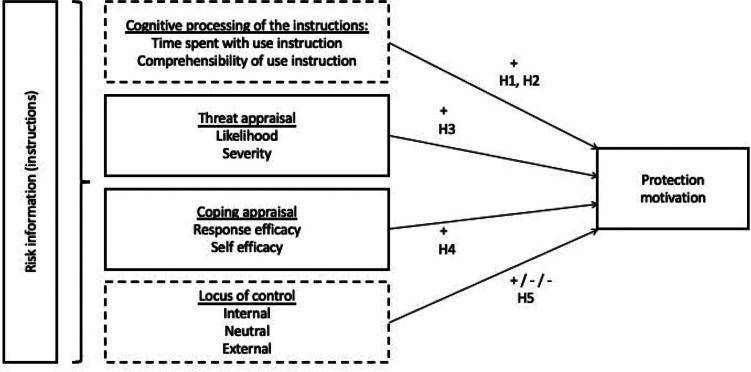
Suggested theoretical background (parts added to the original Protection Motivation Theory in dotted boxes)

The goal of this study was to investigate the attention and comprehension raised by use instructions on biocidal products and to focus on users' willingness to protect the environment within the context of the Protection Motivation Theory, extended with the concept of Locus of Control (Rogers 1975; Rotter 1966). The following hypotheses were derived from this theoretical background and investigated within this study (cf. Figure 1):

- H1: More time spent with the use instructions (i.e., attention) will relate to higher protection motivation.
- H2: The more comprehensible use instructions are rated, the higher the protection motivation.
- H3: A higher threat appraisal (i.e., likelihood and severity of risk) will be associated with higher protection motivation.
- H4: A higher coping appraisal (i.e., response efficacy and self-efficacy) will be associated with higher protection motivation.
- H5: A higher perceived internal locus of control and a lower neutral and external locus of control will be associated with higher protection motivation.

## Methodology

### Study design

This study is based on data gathered within an online survey in early 2022. An online survey was the method of choice in order to reach a large sample of participants from all over Germany. The questionnaire was developed in close collaboration with the German Environment Agency and comprised sections that are not presented in this article, as they were assessed for other purposes. Before the start of data collection, the questionnaire of this online survey was pretested with a convenience sample to ensure comprehensibility and appropriateness of questions (*N* = 28). Target participants for this study were people living in Germany. A quota design with gender, age, and state of residency was applied to ensure a heterogeneous sample and representativeness of the collected data for the German resident population. The participants were recruited with the support of a market research company (https://www.bilendi.de/) and provided informed consent prior to participating in the study.

### Questionnaire

**Fig. 2:**
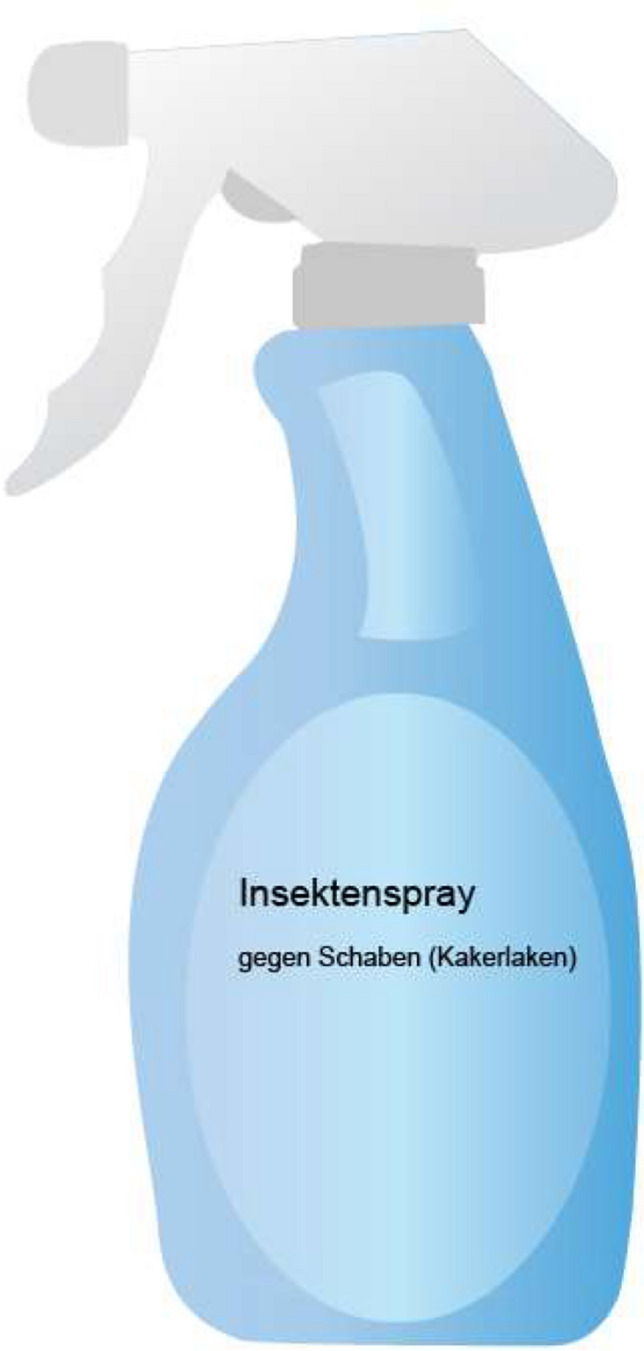
Stylised picture used for the scenario (reads: Insect spray against cockroaches)

The participants were introduced to the following hypothetical purchase scenario and were asked to put themselves in the situation as best as they can: *You notice that there are suddenly a lot of cockroaches in your house or flat. You go to a shop and buy an insect spray.* Then, they were presented with a stylised picture of an insect spray (i.e., the drawing of a blue spray bottle, Fig. 2) and the use instructions below. The use instructions are realistic in the sense that they are comparable in content, complexity, and length to use instructions used for product authorization.

*The application of the insect spray is limited to areas that are not wet cleaned or fully protected from water, such as garages, cellars, attics, cavities, warehouses, electrical service rooms, boiler rooms. The product is applied selectively in cracks and crevices that are present on porous / non-porous surfaces and may provide shelter for crawling insects (cockroaches, ants, silverfish, earwigs) and spiders. On porous surfaces, the effectiveness may be lower. Apply directly into the cracks/crevices. Minimum distance between two pump strokes: 20cm. Application rate: 36 pump blasts/m2 (corresponds to 50 ml product/m2). Max. 72 pump strokes/house and application. Max. 2 applications/year. Wait 4 weeks before the second application. Do not use in areas that are wet cleaned. Do not use in kitchens or bathrooms.*

The measures of interest (i.e., attention, comprehension, protection motivation and predictors) were based on this hypothetical purchase scenario and are introduced subsequently.

#### Attention for the use instruction and further data cleaning

Directly after introducing the scenario and product with the use instructions, the participants were asked whether they had read the instructions (response options: yes or no). The participants that admitted that they had not read the use instructions (*n* = 80, 7%) were excluded from all subsequent analyses. As a proxy measure for attention for the use instructions, the time spent on the page with the use instruction was tracked in seconds. The time spent on the page exhibited large variations (from 2 s to 67 min). Thus, a two-step outlier analysis was conducted. In the first step, participants with z-scores above ± 1.96 were removed from the analyses (*n* = 9). In the second step, the outlier analysis was run again and participants with z-scores above ± 3.29 were removed (*n* = 16).

#### Comprehensibility of use instructions

Comprehensibility of the use instructions was measured with the following three items: 1) These are clear and concrete instructions that are easy to follow (*M* = 3.6, *SD* = 1.2); 2) The text is too complicated for me, and I would not read it (*M* = 2.0, *SD* = 1.2); 3) The text is too long for me, and I would not read it (*M* = 2.2, *SD* = 1.2). The response options ranged from 1 "do not agree at all" to 5 "fully agree." The three items were combined into a single comprehension scale by reverse-coding the second and third item and by taking the mean over all items (α = 0.71). Thus, a higher score on this measure indicates higher comprehensibility of the use instructions on the insect spray.

#### Protection motivation and its predictors

Protection Motivation was measured with a series of eight statements that the participants were asked to agree or disagree with (cf. Table 1). According to the previously shown use instruction, four statements denoted correct behaviour, while the other four statements denoted incorrect behaviour (variables with an asterisk in Table 1). The response options ranged from 1 "do not agree at all" to 5 "fully agree" and participants also had the option to respond that they did not know. The eight items were combined into one final scale by reverse-coding the incorrect behaviour items and taking the mean over all items (α = 0.85). Participants that responded "do not know" to all eight items were coded as missing (*n* = 6). A higher score on the range from 1–5 suggests a higher protection motivation.

**Table 1:** Measurement of protection motivation (*N* = 957, response options ranged from 1 "do not agree at all" to 5 "fully agree")

	*M*	*SD*	% of “do not know”	r_i_
I would only use the insect spray in areas that are protected from water	4.1	1.1	6%	0.58
I would use the insect spray everywhere where cockroaches are present.*	2.6	1.4	4%	0.63
I would use as much insect repellent as necessary, even if it is more than the number of pumps specified in the instructions.*	2.6	1.3	3%	0.68
I would also use the insect spray in the kitchen or bathroom if cockroaches are present there.*	2.4	1.4	6%	0.60
I would pay close attention to how many pumps per m^2^ are given in the instructions and follow them exactly	3.6	1.3	5%	0.56
I would use the insect spray again if there were still cockroaches the next day.*	2.5	1.4	5%	0.56
I would only use the insect spray in cracks or crevices	4.1	1.1	5%	0.53
When applying, I would pay close attention to the minimum distance between two pump strokes	3.6	1.2	5%	0.55

*M* Mean, *SD* standard deviation, *r*_*i*_ corrected item-total correlation, *: reverse-coded items in the final scale

Threat appraisal was measured by asking the participants about the perceived likelihood and severity of harm to human health, to pets and wild animals, and the environment if the insect spray would not be used correctly once (cf. Table 2). Response options for likelihood ranged from 1 “very unlikely,” 2 “rather unlikely,” 3 “rather likely,” to 4 “very likely” and for severity from 1 “not severe at all,” 2 “rather not severe,” 3 “rather severe,” to 4 “very severe;” The scale for threat appraisal was built by taking the mean over the scores for likelihood (α = 0.88) and severity (α = 0.89) for each participant.

**Table 2:** Measurement of threat appraisal (N = 957)

	Likelihood		Severity	
	*M*	*SD*	*M*	*SD*
Harm to human health	2.7	0.8	2.5	0.8
Harm to animal health (own pets or other people’s pets)	3.1	0.8	2.8	0.8
Harm to animal health (wild animals)	2.8	0.9	2.6	0.8
Harm to the environment	3.0	0.8	2.7	0.8

*M* mean, *SD* standard deviation

Coping appraisal was measured with two variables: response efficacy (α = 0.74) and self-efficacy (α = 0.74). Table 3 presents the items of these two scales. The response options ranged from 1 "do not agree at all" to 5 "fully agree." The participants also had the option to indicate that they did not know. To build the scales, the mean was taken over the three respective items (one item was reverse-coded for the self-efficacy scale, denoted by an asterisk in Table 3). Thus, a higher score on these two scales suggests higher response efficacy and self-efficacy respectively.

**Table 3:** Measurement of coping appraisal (N = 957)

	*M*	*SD*	r_i_
Response efficacy			
If you follow the instructions on the insect repellent exactly, you protect your health	3.6	1.1	0.66
If you follow the instructions on the insect repellent exactly, you will ensure its effectiveness	3.8	1.0	0.47
If you follow the instructions on the insect repellent exactly, you protect the environment	3.3	1.2	0.58
Self-efficacy			
I feel able to use the insect spray as instructed	4.2	1.0	0.55
I would find it difficult to follow the instructions on the insect repellent exactly.*	2.5	1.3	0.54
I am sure that I would follow the instructions on the insect spray exactly	3.9	1.1	0.65

*M* mean, *SD* standard deviation, *r*_*i*_ corrected item-total, *: reverse-coded items in the final scale

The internal locus of control focused on health and the environment and was measured with two statements (α = 0.62). Neutral locus of control was measured with two statements on the inherent safety of commercially available products (α = 0.84). External locus of control was measured with two statements on the responsibility of producers (α = 0.80). Table 4 presents the six items. The three scales were built by taking the mean of the two individual variables per person. Higher scores on each of these scales indicate a higher approval of either an internal, neutral, or external locus of control.

**Table 4:** Measurement of locus of control (*N* = 957)

	**M**	**SD**	**r**
Internal locus of control			
It is my responsibility not to harm the environment with the insect spray	4.1	0.9	0.45
It is my responsibility not to damage my health with the insect spray	4.2	1.0	
External locus of control			
It is the responsibility of the manufacturer of insect sprays to produce them in a way that does not harm the environment	3.8	1.1	0.66
It is the responsibility of the manufacturer of insect sprays to produce them in such a way that they do not harm health	3.8	1.1	
Neutral locus of control			
Commercially available insect sprays have been tested by the authorities and are therefore safe for the environment	2.5	1.2	0.72
Commercially available insect sprays have been tested by the authorities and are therefore safe for health	2.6	1.2	

*M* mean, *SD* standard deviation, *r* correlation coefficient, *: reverse-coded items in the final scale

Two control measures were introduced to control for the artificiality of the hypothetical purchase scenario. Namely, the participants were asked how easy it was for them to empathise with the scenario. Additionally, sociodemographic information for all participants was collected for the quota design, namely their gender, age, state of residency.

## Results

### Sample

Initially, *N* = 1088 took part in the survey. This sample was subjected to a thorough data cleaning process, removing *n* = 134 participants in total, i.e., those who had admitted to not having read the use instructions (*n* = 80), who had outliers in the time spent on the page with the use instructions (*n* = 25; see Section "Attention for the use instruction and further data cleaning" for more detailed information), who took less than 5 min for the entire questionnaire (*n* = 20) and who exhibited doubtful response patterns (*n* = 6). This resulted in the final sample of *N* = 957 participants (female: *n* = 488, 51%, male: *n* = 466, 49%; other: *n* = 3, < 1%). The mean age of the participants was *M* = 46 (*SD* = 15, range: 18 to 85 years of age). The distribution of participants according to state corresponds to the national distribution, with more participants from populous states (e.g., North Rhine-Westphalia, Bavaria, and Baden-Wuerttemberg) than from less populous states (e.g., Bremen, Saarland, Hamburg). The participants differed according to their educational background, with *n* = 417 (44%) indicating a low (primary and secondary school diploma, vocational training) and *n* = 540 (56%) indicating a higher education (e.g., “Abitur” or academic degree). Most participants indicated that they could rather (*n* = 524, 55%) or very well (*n* = 299, 31%) empathise with this scenario. Oppositely, *n* = 120 (13%) had some and *n* = 14 (1%) had a lot of difficulties to empathise with the scenario. The participants were asked to indicate if they would use an insect spray in this situation to which most of the participants responded affirmatively (*n* = 706, 74%).

### Attention on use instruction

**Fig. 3:**
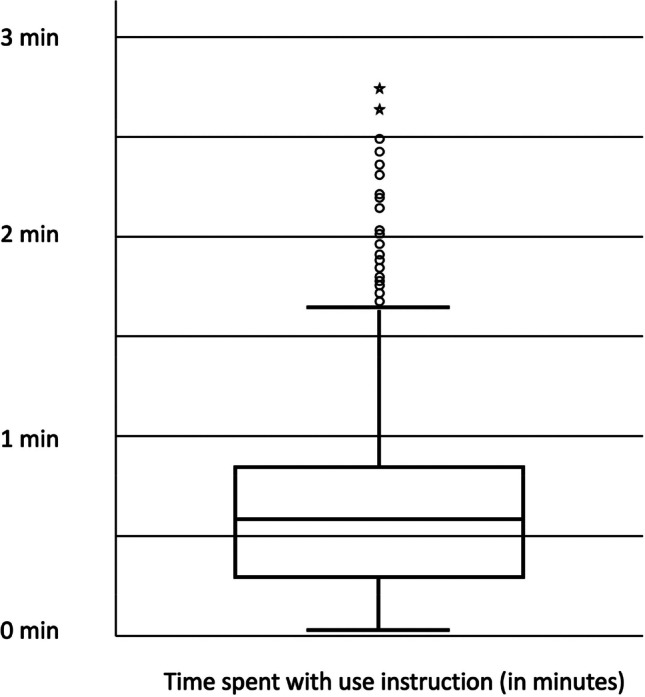
Boxplot of time spent with use instruction (*N* = 957)

Figure 3 shows a boxplot of the time spent with the use instructions. This time varied between 2 s and 2.8 min. The median of the time spent with the use instruction was 35 s and most participants spent less than one minute on the page with the use instruction.

### Comprehensibility of the use instruction

The mean comprehensibility of the use instructions was medium to high (*M* = 3.8, *SD* = 1.0, range 1 to 5) and deviated significantly from the scale's mid-point of 3 (*t*(956) = 25.5,* p* < 0.001, Cohen's *d* = 0.82).

### Protection motivation and its predictors

Table 5 presents the bivariate correlation coefficients for protection motivation and its predictors. All variables were significantly related to protection motivation, apart from external locus of control. Next, a regression analysis was conducted to cover the four aspects attention, comprehension, and the attitudinal predictors of protection motivation (cf. Table 6). The strongest positive associations with protection motivation were found for self-efficacy. Comprehensibility of use instruction, internal locus of control, time spent with use instruction, perceived severity, and response efficacy were also positively associated with protection motivation. Neutral locus of control was negatively associated with protection motivation. No significant associations with protection motivation were observed for perceived likelihood and external locus of control. In summary, participants reported higher protection motivation if they spent more time with the use instruction, perceived it as comprehensible, rated the threat to humans, animals, and the environment as more severe, perceived themselves as responsible (i.e., internal locus of control) and capable (i.e., self-efficacy) of adhering to the use instructions, perceived adherence to the use instructions as effective (i.e., response efficacy) and did not perceive biocidal products as inherently safe (i.e., neutral locus of control).

**Table 5:** Bivariate correlations between protection motivation and predictors (*N* = 951–957)

	Protection motivation	Time spent with use instructions	Compre-hensibility of use instructions	Threat appraisal: Likelihood	Threat appraisal: Severity	Response efficacy	Self-efficacy	Internal locus of control	Neutral locus of control
Protection motivation	-								
Time spent with use instructions	0.25***	-							
Comprehensibility of use instructions	0.45***	0.22***	-						
Threat appraisal: Likelihood	0.26***	0.01	0.10**	-					
Threat appraisal: Severity	0.24***	-0.04	0.06	0.69***	-				
Coping appraisal: Response efficacy	0.26***	0.10**	0.29***	0.03	-0.01	-			
Coping appraisal: Self-efficacy	0.59***	0.17***	0.53***	0.22***	0.18***	0.36***	-		
Internal locus of control	0.38***	0.22***	0.25***	0.23***	0.18***	0.33***	0.38***	-	
Neutral locus of control	-0.19***	-0.17***	-0.03	-0.18***	-0.10**	0.29***	-0.02	-0.13***	-
External locus of control	0.04	0.11**	0.01	0.07*	0.10**	0.03	0.09**	0.10**	0.12***

***: p < 0.001, **: p < . 01. *: p < *0.05*

**Table 6:** Linear regression with protection motivation as dependent variable (*N* = 951)

	*B* [95% CI]	β	*t*	*p*
Constant	0.6 [0.3, 1.0]		3.7	< 0.001
Time spent with use instruction	0.0 [0.0, 0.0]	0.10	3.8	< 0.001
Comprehensibility of use instruction	0.1 [0.1, 0.2]	0.16	5.4	< 0.001
Threat appraisal: Likelihood	0.1 [0.0, 0.1]	0.05	1.4	0.152
Threat appraisal: Severity	0.1 [0.0, 0.2]	0.09	2.8	0.006
Coping appraisal: Response efficacy	0.1 [0.0, 0.1]	0.08	2.7	0.007
Coping appraisal: Self-efficacy	0.4 [0.3, 0.4]	0.40	12.9	< 0.001
Internal locus of control	0.1 [0.0, 0.2]	0.10	3.4	< 0.001
Neutral locus of control	-0.1 [-0.2, -0.1]	-0.15	-5.4	< 0.001
External locus of control	0.0 [-0.1, 0.0]	-0.02	-0.8	0.429

R^2^ = 0.45, *F(9, 941)* = *84.2, p* < *0.001*

## Discussion

### Summary of insights on attention, comprehension, and willingness to protect the environment

In the present study, the attention for and comprehension of use instructions on biocidal products, and the willingness to protect the environment were investigated. The Protection Motivation Theory and the concept of Locus of Control served as theoretical backdrop (Rogers 1975; Rotter 1966). Based on the variance that could be explained by the adapted theory, it can be concluded that the PMT and its components could be a useful model for future inquiries into consumers’ attention for and protection motivation elicited by information on biocidal products. Subsequently, the insights generated on attention, comprehension and willingness to protect the environment are outlined and embedded into prior insights on chemical risk perception.

Despite the length of the realistic use instructions, the participants in our study spent relatively little time with them. So far, little has been known about the preconditions for the implementation of risk mitigation measures by biocidal product users. Prior insights into individual, product and situational aspects that lead to lower or higher attention for or comprehension of warning labels on hazardous chemicals could potentially be transferred to use instructions on biocidal products (Buchmueller et al. 2022; Geuens et al. 2021),. However, the degree of transferability needs to be investigated further as hazard warnings, such as the GHS pictograms, and risk mitigation measures (i.e., specific use instructions) provide different information. GHS labels symbolise the intrinsic properties of the contained ingredients or substance in a biocidal product, and provide general information on e.g., use or disposal. In contrast, risk mitigation measures are the result of a complex risk assessment process and provide information for a specific use case of a biocidal product (e.g., indoor or outdoor use). Hence, it may be possible that a product does not feature a GHS pictogram but rather specific use instructions as risk mitigation measure. This article focused specifically on use instructions (not warnings) to investigate their effect on consumers’ perceptions and behaviours. Similar aspects might contribute to the amount of attention that is paid to warning labels and use instructions (e.g., higher risk perception might lead to higher attention for information on the product in general). However, the transferability of the insights from warning labels to use instructions needs to be tested in the future.

On the individual level, it has been suggested that risk awareness and aversion will likely increase the attention given to warning information and use instructions (Bianco et al. 2008; Buchmueller et al. 2020; Wieck et al. 2018a). Simply put, users that are aware of the potential risks of a biocidal product to health and the environment will spend more time reading the use instructions to ensure safe handling. On the product level, several product attributes were identified in prior studies that either heightened or reduced the initial risk perception of a chemical household product (Basso et al. 2016; Bearth et al. 2022; Bearth et al. 2017; Bearth and Siegrist 2019). These insights could potentially be transferred to biocidal products and the context of use instructions. Products with coloured and prominently placed warning labels elicit higher risk perception compared to products without warning labels (Boelhouwer and Davis 2010; Buchmueller et al. 2022; Wogalter et al. 2002). If a biocidal products seems highly effective in completing a specific task, it will be perceived as more dangerous and use instructions will elicit more attention (Bearth et al. 2017). A biocidal product will be perceived as less risky if it elicits high perceived naturalness (i.e., ecological products, green packaging) (Bearth et al. 2017; Bearth and Siegrist 2019). On the situational level, distractions, and resource restraints (i.e., time, motivation) might contribute to less attention for use instructions independent of the initial risk perception.

The comprehensibility of the use instruction featuring 124 words was rated in a positive light by many participants. A realistic use instruction was used that can also be found on biocidal products available on the market. Therefore, this finding suggests carefully optimistic conclusions that limited comprehension is not the most important contributor to the incorrect use of biocidal products. Likely, more important is the lack of attention for use instructions. However, conclusions regarding comprehension are limited in this study, as comprehension was not tested but rather assessed in self-report. Future studies should investigate this point further by testing comprehension of use instructions instead of asking for comprehension in self-report.

Oppositely, various insights can be gained from the results on protection motivation and predictive psychological factors. Self-efficacy, meaning the perceived ability to adhere to the instructions, was most strongly related to protection motivation. Self-efficacy will likely be higher if instructions are comprehensible to the user, if instructions are practicable, and users have all required material at hand (i.e., dosing instruments, protective gear). Thus, self-efficacy could be supported by optimising comprehensibility (e.g., avoiding ambiguous terms or quantifications, giving examples) and by providing additionally needed material together with the biocidal product. This point is further strengthened by the comparably high effect size of comprehensibility, meaning that protection motivation was higher if self-reported comprehensibility was rated higher. Perceived threat was significantly related to protection too but only severity ratings. This makes sense, as prior literature suggests that people tend to focus more on hazard rather than risk when judging chemical risk (Bearth et al. 2019; Kraus et al. 1992; Saleh et al. 2019). Thus, adherence to use instructions could potentially be increased through information provision with a focus on the severity of outcomes for humans, animals, and the environment. These informational efforts would also contribute to a higher initial risk perception of biocidal products under the condition that a product is recognised as biocidal (Wieck et al. 2018a, 2018b). This could be paired with information that increase the perceived responsibility of the user to avoid harming health and the environment when using biocidal products (i.e., internal locus of control). Important antagonists to these informational or educational efforts are a lack of perceived responsibility and efficacy of following the use instructions. If users perceive all commercially available biocidal products as safe, they will not perceive themselves responsible to protect health and the environment. Similarly, if users do not see a direct link between adhering to the use instructions and avoiding environmental harm, they will likely exhibit lower protection motivation.

### Implications for an appropriate risk management of biocidal products

The overall objective of risk mitigation measures is to ensure the safe use of a biocidal product. Risk mitigation measures are the result of a complex risk assessment process determining safe use conditions for authorisation. To make risk mitigation measures comprehensible and practicable for users, they are often supplemented by use instructions that need to be followed to minimise the risks to an acceptable level. A safe use of biocidal products is assumed only if risk mitigation measures are implemented correctly. In contrast to other chemical products (e.g., plant protection products), a substantial part of the biocidal products on the market are intended for use by consumers in their private homes. Hence, the determination of the use conditions for consumer products during authorisation requires special attention as it is assumed that this user category applies biocidal products only sporadically for a specific problem and has no routine expertise or training in handling such products. Consequently, to prevent potential risks to the environment, it is of utmost importance that instructions are easy to comprehend and easy to implement. This makes the question of the factors that might predict users' willingness to implement the use instructions highly relevant for regulation. The insights from this study that 1) self-efficacy and comprehensibility of use instructions and 2) perceived severity are strongly related to protection motivation highlights two weak points in the current authorisation practice of biocidal products. These are outlined below and suggestions for improvements are made.:

First, in light of the importance of self-efficacy (i.e., perceived ability to adhere to instructions) and comprehensibility (i.e., how easy the use instructions are to understand), there is a lack of harmonised approaches and guidance for the assignment of use instructions and risk mitigation measures. Biocides are a highly diverse group of chemicals with various product types, application methods and different target organisms. Consequently, there are many technical aspects potentially relevant to be considered for risk management. In practice, risk mitigation measures are often created and assigned on a case-by-case basis with focus on regulatory aspects or exposure assessment. As a result, the instructions are often rather long, complex, and technical (e.g., using terms from emission scenario documents normally used for theoretical model calculations). In a study investigating the perception of biocidal products by consumers, 42.6% of interviewees indicated that they follow the instructions only sometimes, mainly if they comprehend the instructions (29.5%) or are able to implement them practically (20.5%) (Wieck et al. 2018a, 2018b). Hence, it would be important to systematically assess whether instructions used to minimise environmental risks are comprehensive and practicable for consumers and based on this streamline the processes and harmonize the decision to ensure equal treatment of biocidal products. The social sciences could support this process by first, providing guidance on how to evaluate the comprehensibility and practicability of use instructions and risk mitigation measures and second, transferring established strategies for risk communication from other areas of interest (e.g., Edworthy et al. 2004; Maghroudi et al. 2021; Wogalter et al. 2002). Some specific strategies for biocidal products could be 1) to provide application rates that are easier to implement (e.g., pump strokes of a spray instead of application rates in g/m^2^, inclusion of dosing aid), 2) to adjust the package size (e.g., reduction of the amount of unused product, which will turn into hazardous waste), or 3) to avoid ambiguous terminology and provide examples (e.g., do not use the product when it is windy, instead of avoid wind drift).

Second, the perceived threat was related to protection motivation, which points at another weak point for risk management mainly relying on use instructions on the label: It only works if consumers pay attention to the instructions before use. For the regulation of biocidal products this constitutes a problem, as a safe use is a pre-condition for authorisation that is only assumed if the instructions are followed. While, consumers do not necessarily need to know what the theoretical term biocide means, it is important that they are aware about the potential risks associated with the use of biocidal products on the market and the importance to adhere to the use instructions on the label. Already when drafting the BPR, legislators have been aware of this problem: article 17 (5) of the BPR requires EU Member States to provide the public with information on the benefits and risks of biocides and to inform about ways to minimize their use (European Union 2012). To fulfil this mandate, some member states offer information on biocidal products within their national information offers (Wirth et al. 2022), e.g. the German Environment Agency (Umweltbundesamt—UBA) is hosting the information portal “Biozid-Portal”. Moreover, article 69 (2) of the BPR sets out that labels must not be misleading concerning the risks of biocidal products and must not contain indications such as ‘low-risk biocidal product’, ‘non-toxic’, ‘harmless’, ‘natural’, ‘environmentally friendly’, ‘animal friendly’ or similar indications.” (European Union 2012). In practice, this article has received too little attention so far and should be implemented more consequently by both applicants and member states when authorizing biocidal products.

However, whether these efforts to improve risk awareness are sufficient to guarantee an effective risk management of consumer products in the long run, requires closer investigation: Although the German Environment Agency is hosting the “Biozid-Portal” since 2010, a survey conducted in Germany in 2015 showed that 39% of interviewees had not heard of the term biocide. Moreover, 52.4% indicated to always read the instructions and 42.7% only sometimes (Wieck et al. 2018a, 2018b). Hence, besides the above easy-to-consider suggestions, there is also a need to systematically address the need to establish criteria for the regulatory decision under which use conditions products (e.g., level of details, additionally needed equipment or expert knowledge) are no longer suitable for consumers, but should be restricted for use by professionals.

### Study limitations and insights for future research

A major limitation of this study is the use of self-report that might trigger socially desirable responses. The effect of social desirability becomes particularly apparent in the comparison of the self-reported attention and the time spent on the page with the use instructions. Socially desirable responses might imply that the attention, comprehension, and protection motivation are overestimated in this study compared to motivation and behaviour using biocides in the home. Regarding attention, future studies could further the insights gathered in this study by applying observational measures, such as reading time, or eye tracking to determine which areas on biocidal products receive more attention (Buchmueller et al. 2022). Regarding comprehension, a knowledge test after exposing participants to use instructions could garner additional insights whether the use instructions were understood in the intended way. Regarding protection motivation, insights into actual behaviour when using biocidal products are needed by for example observing participants while the use biocidal products. Particularly, contextual factors (e.g., distractions, poor inhibitory control, lack of effectiveness upon initial use of biocidal product) might decrease protection motivation and thus, lead to unsafe behaviour. Unfortunately, observational methods are also subjected to biases, as participants are aware of being observed and might exhibit different behaviour than when they are unobserved. Creative ways to reduce the impact of these biases, such as hiding the true purpose of the observation or reducing the experienced observation, are needed for the future.

### Conclusion

To conclude, this study offers relevant insights for the risk management of biocidal products. First and foremost, it stresses that most participants spent relatively little time with the use instruction. Therefore, it is important to limit design features on biocidal product packaging that reduce users' risk awareness, such as article 69 (2) of the BPR foresees. Various design features might potentially be misleading, such as colour, production origin or imagery (e.g., Basso et al. 2016; Bearth et al. 2022, 2017; Buchmueller et al. 2020; Marcell and Isaacson 2015). An extensive knowledge base on these features is available (i.e., lower risk awareness for ecological products or products that signal low risk or effectiveness) and insights can be transferred to the regulation of biocidal products. The study also highlights various factors that are closely related to protection motivation, such as self-efficacy (i.e., feeling able to protect the environment through understandable and practicable use instructions) and perceived threat (i.e., feeling that biocidal products are a threat to human health or the environment). These could be used to conceptualise interventions to encourage users of biocidal products to protect the environment by spending more time and energy reading the labels and implementing the instructions. Second, the establishment of use instructions suitable and comprehensible for consumers should receive more attention in the future. The findings of this study, albeit based on self-report, indicate that limited comprehension is not the most important obstacle for the successful risk management of biocidal products for non-professional users. However, due to the methodological limitations associated with self-report, verification of the results using other methods and possibly including instructions for health protection are needed. It might be worthwhile to also consult literature from other products featuring use instructions (i.e., medicine, cleaning products). Finally, criteria should be established for the regulatory decision under which use conditions products are no longer suitable for consumers but should be restricted for use by professionals.

## Data Availability

The data that support the findings of this study are available from the corresponding author upon request.
